# Evidence of a role for CutRS and actinorhodin in the secretion stress response in *Streptomyces coelicolor* M145

**DOI:** 10.1099/mic.0.001358

**Published:** 2023-07-07

**Authors:** Thomas C. McLean, Ainsley D. M. Beaton, Carlo Martins, Gerhard Saalbach, Govind Chandra, Barrie Wilkinson, Matthew I. Hutchings

**Affiliations:** ^1^​ Department of Molecular Microbiology, John Innes Centre, Norwich, Norwich Research Park, NR4 7UH, UK; ^2^​ Department Biochemistry and Metabolism, Proteomics Facility, John Innes Centre, Norwich, Norwich Research Park, NR4 7UH, UK

**Keywords:** *Streptomyces*, antibiotics, actinorhodin, secretion stress, two-component system, protein secretion, proteomics

## Abstract

CutRS was the first two-component system to be identified in *Streptomyces* species and is highly conserved in this genus. It was reported >25 years ago that deletion of *cutRS* increases the production of the antibiotic actinorhodin in *Streptomyces coelicolor *. However, despite this early work, the function of CutRS has remained enigmatic until now. Here we show that deletion of *cutRS* upregulates the production of the actinorhodin biosynthetic enzymes up to 300-fold, explaining the increase in actinorhodin production. However, while ChIP-seq identified 85 CutR binding sites in *S. coelicolor* none of these are in the actinorhodin biosynthetic gene cluster, meaning the effect is indirect. The directly regulated CutR targets identified in this study are implicated in extracellular protein folding, including two of the four highly conserved HtrA-family foldases: HtrA3 and HtrB, and a putative VKOR enzyme, which is predicted to recycle DsbA following its catalysis of disulphide bond formation in secreted proteins. Thus, we tentatively propose a role for CutRS in sensing and responding to protein misfolding outside the cell. Since actinorhodin can oxidise cysteine residues and induce disulphide bond formation in proteins, its over production in the *∆cutRS* mutant may be a response to protein misfolding on the extracellular face of the membrane.

## Impact statement

It is important for bacteria to sense their environment in order to survive in dynamic and changing niches. Two-component systems (TCS), typically consisting of a membrane-bound histidine kinase and a cognate response regulator, are a common example of these types of sensors. *Streptomyces* spp. are Gram-positive bacteria with complex life cycles, occupying diverse environmental niches and with the ability to produce a wide array of specialized metabolites, including antibiotics that are useful to humans. CutRS was the first two-component system to be identified in the genus *Streptomyces* and deletion of the *cutRS* genes from the *S. coelicolor* genome led to abnormally high levels of the redox active antibiotic actinorhodin being produced. In this work we have further characterized the mechanisms that underpin this relationship. It was shown, by identifying the genome locations where CutR (the response regulator) binds, that the direct effect of this TCS is not on the actinorhodin biosynthetic gene cluster but rather genes involved in secretion stress responses. This is interesting as it suggests that actinorhodin may be produced as a participant in the secretion stress response. Furthermore, our work highlights the importance of understanding the regulation of bacterial specialized metabolite production for the purposes of new molecule discovery and industrial production.

## Data Summary

The authors confirm all supporting data, code and protocols have been provided within the article or through supplementary data files.

## Introduction

*Streptomyces* species are ubiquitous soil bacteria that play important roles in the turnover of organic material in the soil and form beneficial interactions with plants and insects [1, 2]. They are studied due to their complex developmental life cycles, which include hyphal growth, sporulation, and exploration [3], and because they make a diverse array of bioactive specialized metabolites, many of which are used clinically as antibiotics [4]. Understanding the control of the complex *Streptomyces* life cycle in response to nutritional and other environmental cues is a major challenge and is important because the progression of the life cycle into sporulation is linked to the production of antibiotics [5]. With 97 % of their specialized metabolite biosynthetic gene clusters (BGCs) yet to be matched to molecules, understanding the environmental signals and signalling pathways that control the expression of these cryptic gene clusters is expected to lead to the discovery of new molecules, including new antibiotics [6].

One of the major ways in which *Streptomyces* bacteria sense and respond to their environment is via two-component systems (TCS), which consist of a sensor histidine kinase (SK), which usually spans the cell membrane, and a cytoplasmic response regulator (RR) that typically modulates target gene expression [7]. The SK senses a signal outside the cell, autophosphorylates its cytoplasmic kinase domain, and the RR then catalyses the transfer of that phosphate to its own receiver domain to switch on its DNA binding activity. Many of the TCS that have been characterized in the genus *Streptomyces* have been shown to either directly or indirectly affect antibiotic production, but relatively few systems have been characterized in detail [8]. We recently compared 93 complete *Streptomyces* genome sequences and identified 15 conserved TCS encoded by these bacteria, one of which is called CutRS [9]. This was the first TCS to be identified in the genus *Streptomyces* >25 years ago and the genes (a complete *cutR* and partial *cutS*) were first cloned and sequenced because a mutation in *cutR* was thought to suppress a mutation in *melC1*, the gene required for the assembly of the copper containing tyrosinase MelC2. The TCS was named CutRS because it was thought to be involved in regulating copper metabolism [10]. However, a later study, which cloned and sequenced the entire *cutRS* operon and compared it to the *cutRS* genes in the *melC1* suppressor strain, revealed there were no mutations and they concluded that the suppressor lies outside of *cutRS* [11]. This work also reported that deletion of *cutRS* in *Streptomyces coelicolor* or *Streptomyces lividans* increases production of the blue-pigmented antibiotic actinorhodin and the authors concluded that CutR represses actinorhodin biosynthesis. Despite this pioneering early work, the function of CutRS, its target genes, and its role in controlling antibiotic production remain unknown.

In this work we set out to investigate the role of CutRS in controlling actinorhodin production in *S. coelicolor* M145. We confirmed that deletion of *cutRS* increases actinorhodin production in this species and report that an *S. coelicolor ∆cutRS* mutant can be complemented in trans by its own *cutRS* operon and those from the distantly related species *Streptomyces formicae* [12] and *Streptomyces venezuelae* [13], supporting the hypothesis that CutRS structure and function are highly conserved in this genus. We then used ChIP-seq to identify the conserved CutR regulon in *S. coelicolor* using both the native *cutRS* (*SccutRS*) and the heterologously expressed *S. venezuelae cutRS* (*SvcutRS*) genes. Intriguingly, we found that DNA binding by ScCutR and SvCutR in *S. coelicolor* is dependent on the addition of glucose to the growth medium and we identified 16 binding sites that are shared by both CutR proteins on the *S. coelicolor* genome. Through quantitative and comparative tandem-mass tagging (TMT) proteomics we show that CutR directly controls the production of *S. coelicolor* HtrA3 and HtrB, which are two of the four conserved HtrA (high temperature requirement) proteins in *Streptomyces* species. This family of proteins typically act as chaperones and quality control proteases for secreted proteins and their production is controlled in response to secretion and/or cell envelope stress [14]. CutR also directly controls the production of a putative vitamin K epoxide reductase (VKOR) enzyme SCO1507, a member of group of enzymes that perform the same function as DsbB, i.e. they recycle DsbA following its catalysis of disulphide bond formation in secreted proteins [15]. Thus, we propose that CutRS has a role in the secretion stress response in *S. coelicolor *, which is triggered when proteins fold incorrectly following export through the general secretion pathway (Sec). Whilst the actinorhodin biosynthetic enzymes are increased up to 300-fold in the *∆cutRS* mutant, the over-production of actinorhodin must be indirect because CutR does not bind within the actinorhodin BGC. Since actinorhodin is redox active and can oxidise cysteine residues [16], it is possible this specialized metabolite is produced to assist the folding of secreted proteins under times of stress.

## Methods

### Strains, plasmids and primers

All bacterial strains used in this work are listed in Table 1 and the plasmids used in this work are listed in Table 2. Those that were generated for this work were constructed as follows: DNA fragments containing 25–35 nucleotide overlapping regions were assembled into digested DNA vectors using the exonuclease-based Gibson Assembly (NEB). A standard 3 : 1 ratio of insert to vector was used and assembly performed at 50 °C for 1 h in a thermocycler. Primers used in this work are listed in Table 3.

**Table 1.** Bacterial strains used in this work

Bacterial strain	Description	Supplier and/or reference
*E. coli* DH5alpha	*E. coli* strain used for cloning	ThermoFisher
*E. coli* Top10	F– *mcr*A Δ(*mrr-hsdRMS-mcrBC*) Φ80*lac*ZΔM15 Δ*lac*X74 *rec*A1 *ara*D139 Δ(*ara* leu) 7697 *gal*U *gal*K *rps*L (StrR) *end*A1 *nup*G	Invitrogen
*E. coli* ET12567/pUZ8002	A methylation deficient (*∆dcm∆dam*) strain of *E. coli* containing the driver plasmid pUZ8002 for conjugation to *Streptomyces* species.	John Innes Centre [20, 21]
* S. coelicolor* M145	Wild-type *S. coelicolor* A3(2) lab strain mutagenized to lose the plasmids SCP1 and SCP2	John Innes Centre [20, 21]
* S. coelicolor* M145 *∆cutRS*	* S. coelicolor* M145 with an unmarked, in-frame deletion to remove the *cutRS* genes	This work
* S. coelicolor* M145 *∆cutRS* +pTCM003	*∆cutRS* complemented in trans with the *SccutRS* operon under the ermE* promoter	This work
* S. coelicolor* M145 *∆cutRS* +pTCM002	*∆cutRS* complemented in trans with the *SvcutRS* operon under the ermE* promoter	This work
* S. coelicolor* M145 *∆cutRS* +pTCM004	*∆cutRS* complemented in trans with the *S. formicae cutRS (SfcutRS*) operon under the ermE* promoter	This work
* S. coelicolor* M145 *∆cutRS* +pTCM007	*∆cutRS* complemented in trans with the *SccutRS* operon encoding C-terminally 3xFlag tagged CutR under its native promoter	This work
* S. coelicolor* M145 *∆cutRS* +pTCM008	*∆cutRS* complemented in trans with the *SvcutRS* operon encoding C-terminally 3xFlag tagged CutR under its native promoter	This work

**Table 2.** Plasmids used in this work

Plasmid	Description	Resistance	Reference
pIJ10257	oriT, ΦBT1 attB-int, hygR, ermEp*	HygR,	John Innes Centre [28]
pSS170	*oriT, ΦBT1 attB-int, hygR*	HygR,	Gift from Susan Schlimpert, JIC.
pTCM002	pIJ10257 +*SvcutRS*	HygR,	This work
pTCM003	pIJ10257 +*SccutRS*	HygR,	This work
pTCM004	pIJ10257 +*SfcutRS*	HygR,	This work
pTCM007	pSS170 +*SccutRSp-SccutRS* 3xFLAG	HygR,	Genewiz. This work
pTCM008	pSS170 +*SvcutRSp-SvcutRS* 3xFLAG	HygR,	Genewiz. This work
pCRISPomyces-2	*AprR, oriT, reppSG5(ts), oriColE1, sSpcas9,* synthetic guide RNA cassette	AprR	[17] RRID:Addgene_61737

**Table 3.** Primers used in this work

Primer	Primer sequence (5’−3’)	Function
pSS170_seqF	GGTCTGACGCTCAGTGGAAC	Forward primer to sequence pSS170 vector inserts
pSS170_seqR	GCTCAGTATCACCGCCAGTG	Reverse primer to sequence pSS170 vector inserts
pIJ10257_seqF	GATCTTGACGGCTGGCGAGAG	Forward primer to sequence pIJ10257 vector inserts
pIJ10257_seqR	GCGTCAGCATATCATCAGCGAGC	Reverse primer to sequence pIJ10257 vector inserts
pCRISP_F	AGGCTAGTCCGTTATCAACTTGAAA	Forward primer to sequence pCRISPomyces-2 editing template inserts
pCRISP_R	TCGCCACCTCTGACTTGAGCGTCGA	Reverse primer to sequence pCRISPomyces-2 editing template inserts
pCRISP_spacer	ATACGGCTGCCAGATAAGGC	Primer to sequence pCRISPomyces-2 sgRNA inserts
TCMP038	GCAAGATCGTCGAACTGGG	Forward primer to amplify the *SccutRS* operon
TCMP039	GCTCGCTGCGTTCGTTGG	Reverse primer to amplify the *SccutRS* operon
TCMP040	TCGGTTGCCGCCGGGCGTTTTTTATCTAGAGACGGACTTCGGAGCGTAGG	Forward primer to amplify 1 kb *SccutRS* homology arm - left
TCMP041	AGGACAAGCGACGTGCGCGTACTCGTCGTCGGGCTCGTGATGCGCGTGAC	Reverse primer to amplify 1 kb *SccutRS* homology arm - left
TCMP042	CAGACGGGGAGCGTCACGCGCATCACGAGCCCGACGACGAGTACGCGCAC	Forward primer to amplify 1 kb *SccutRS* homology arm - right
TCMP043	GCGGCCTTTTTACGGTTCCTGGCCTCTAGACCAGGTCGATGATCCGGTCG	Reverse primer to amplify 1 kb *SccutRS* homology arm - right
TCMP044	ACGCCAGCGTGAGCCTTATCCGGA	Forward primer for *SccutRS* sgRNA
TCMP045	AAACTCCGGATAAGGCTCACGCTG	Reverse primer for *SccutRS* sgRNA
TCMP046	AAAATCTCGCATTCCCGCTC	Primer to sequence the *SccutRS* homology arms central junction
TCMP047	CGTCTTCGAACCCCTCCAGC	Forward primer to confirm deletion of *cutRS* in *S. coelicolor *
TCMP048	CGCTCAAAGCGAACATCCTCG	Reverse primer to confirm deletion of *cutRS* in *S. coelicolor *
TCMP049	GAGAGACATATGCGTGTACTCGTCGTCGAGG	Forward primer to amplify the *SfcutRS* operon
TCMP050	CTCTCTAAGCTTTCAGAGGGGCAGGGTGACAC	Reverse primer to amplify the *SfcutRS* operon
SCO3977 FWD	CCAACGGCAAGAAGTACGA	Forward primer for amplification of *sco3977* (*htrA3*)
SCO3977 FWD	CCAACGGCAAGAAGTACGA	Reverse primer for amplification of *sco3977* (*htrA3*)
SCO4157 FWD	GTCGTTCGCGTCACCTT	Forward primer for amplification of *sco4157* (*htrB*)
SCO4157 REV	GTCCACGACGACGTACAA	Reverse primer for amplification of *sco4157* (*htrB*)
SCO5820 FWD	AGCTGCACTCCGTTCTC	Forward primer for amplification of *sco5820* (*hrdB*)
SCO5820 REV	CGTACACCTTGCCGATCT	Reverse primer for amplification of *sco5820* (*hrdB*)

### Growth media and conditions

The media used are listed in Table 4. The antibiotics used for selection of plasmid carriage were 50 µg ml^−1^ of apramycin and 50 µg ml^−1^ hygromycin as indicated by the resistance markers listed in Table 2. After conjugation, 25 µg ml^−1^ of nalidixic acid was used to kill *E. coli* and select for *Streptomyces* species.

**Table 4.** Growth media used for bacterial culturing (from http://actinobase.org). N/A indicates no media pH buffering was performed.

Media	Recipe (per litre)	Notes	Water	pH
SFM	20 g soya flour, 20 g mannitol, 20 g agar		Tap	n/a
MYM+TE	4 g maltose, 4 g yeast extract, 10 g malt extract, 2 ml trace element solution, +/- 20 g agar	Trace elements solution: ZnCl2: 40 mg/L, FeCl3.6H2O: 200 mg/L, CuCl2.2H2O: 10 mg/L, MnCl2.4H2O: 10 mg/L, Na2B4O7.10H2O: 10 mg/L, (NH4)6Mo7O24.4H2O: 10 mg/L	50 : 50 tap:deionised	7.3
LB	10 g tryptone, 5 g yeast extract, 10 g NaCl +/-20 g agar	NaCl excluded for hygromycin selection	Deionised	n/a
DNA/DNAD	4 g Difco Nutrient, Broth powder, 10 g agar	100 ml/L 40 % glucose solution added after autoclaving for DNAD	Deionised	n/a
2X YT	31 g 2 X YT Broth (Formedium)		Deionised	7.4

### CRISPR/Cas9 mediated deletion of *cutRS* in *Streptomyces coelicolor*

This methodology for scar-less gene deletion and genome editing in *Streptomyces* uses CRISPR/Cas9 as previously described [17]. Using CRISPy-web (https://crispy.secondarymetabolites.org, RRID:SCR_017970) a 20 nucleotide protospacer was designed to target the region of interest via a synthetic guide RNA (sgRNA). In total, 24 nucleotide primers were designed to construct this protospacer containing 5′ BbsI sticky ends. The pair of oligonucleotides were resuspended to 100 µM in deionised water (dH_2_O) before 5 µl of both were mixed in 90 µl 30 mM HEPES pH 7.8. The mixture was heated to 95 °C for 5 min before ramping to 4 °C at a rate of 0.1 °C per second. Golden gate [18] assembly was used to insert the annealed protospacer into pCRISPomyces-2 at the BbsI site and the resulting vector heat shocked into chemically competent *E. coli* Top10 (Invitrogen) before plating on selective LB containing IPTG and X-Gal. White colonies were picked into 10 ml selective LB for overnight incubation. Following plasmid preparation vectors were sequence confirmed and digested with XbaI. Gibson Assembly [19] was used to assemble two 1 kb PCR-amplified homology repair templates, matching regions adjacent to the target region, into the vector. The sequence-confirmed final vector was moved into *Streptomyces* via *E. coli* ET12567/pUZ8002 conjugation and apramycin-resistant colonies were PCR-screened for desired mutations. Finally, the mutants were passaged for multiple generations at 37 °C to facilitate the loss of the temperature-sensitive pCRISPomyces-2.

### Conjugation of plasmids to *S. coelicolor* via *E. coli* ET12567/pUZ8002

Single colonies of *E. coli* ET12567/pUZ8002 containing the desired target vector were picked from selective LB agar plates and incubated overnight in 10 ml selective LB broth, shaking at 220 r.p.m. The overnight culture was sub-cultured in 50 ml selective LB broth and grown to OD_600_ 0.6. The cultures were washed with 10 ml ice-cold LB broth twice by centrifugation to remove the residual antibiotics and finally resuspended in 1 ml ice-cold LB broth. Then, 20–50 µl of *Streptomyces* spores were suspended in 500 µl 2xYT and heat shocked at 50 °C for 10 min. Overall, 500 µl of the *E. coli* suspension was combined with the spore suspension, mixed by inversion and pelleted at 13 000 r.p.m. for 2 min. The supernatant was removed, and the pellet resuspended in 150 µl residual supernatant. Serial dilutions were performed and plated on SFM agar +10 mM MgCl_2_. Plates were incubated at 30 °C for 16–20 h and subsequently overlaid with 1 ml sterile dH_2_O containing 1.25 mg of the selective antibiotic and 0.5 mg nalidixic acid. Once dried, the plates were incubated at 30 °C for 3–7 days until colonies appeared. Colonies were then re-streaked onto SFM agar containing selective antibiotics (including nalidixic acid) at least once before being plated for spore preparation [20, 21].

### Tandem-mass-tagging proteomics

*S. coelicolor* colonies were grown on cellophane covered DNA or DNAD agar plates as triplicate spots from 5 µl spores, all in duplicate. After 5 days at 30 °C the mycelium was scraped into a 15 ml Falcon tube and resuspended in 10 ml cell lysis buffer [50 mM TEAB buffer pH 8.0, 150 mM NaCl, 2 % SDS, EDTA-free protease inhibitor, PhosSTOP phosphatase inhibitor (Sigma Aldrich)]. The suspension was disrupted via French press three times before being boiled for 10 min. Samples were sonicated at 50 kHz four times for 20 s per cycle and then pelleted at 4 000 r.p.m. for 30 min. Protein concentration was determined using the BCA assay and 1 mg of protein from each sample transferred to a fresh 15 ml Falcon tube. Four volumes of methanol were added and vortexed thoroughly before one vol of chloroform was added and vortexed thoroughly. Three volumes dH_2_O was added, vortexed thoroughly, and spun for 10 min at 4 000 r.p.m. The upper layer was carefully discarded, four volumes of methanol were added and vortexed thoroughly. The samples were spun for 20 min at 4 000 r.p.m. before aspirating the supernatant.

Pellets from the protein extraction were washed with acetone and dissolved in 50–100 µl of 0.2 M EPPS buffer pH8 (Merck) with 2.5 % sodium deoxycholate (SDC; Merck). After quantification by BCA assay, 100 µg of protein was reduced and alkylated with 1,4-dithiothreitol and iodoacetamide and digested with trypsin according to standard procedures. After digestion, the SDC was precipitated by adjusting to 0.2 % TFA, and the clarified supernatant subjected to C18 solid phase extraction (SPE; OMIX tips; Agilent). TMT labelling was performed using a Thermo TMT16plex kit according to the manufacturer’s instructions with slight modifications; the dried peptides were dissolved in 90 µl of 0.2 M EPPS buffer pH8/10 % acetonitrile, and 250 µg TMT16plex reagent dissolved in 22 µl of acetonitrile was added. Samples were assigned to the TMT channels in an order avoiding channel leakage between different samples, as detailed by [22].

After labelling, aliquots of 1.7 µl from each sample were combined and analysed on the mass spectrometer (detailed below) to check labelling efficiency and estimate total sample abundances. The sample aliquots were then combined correspondingly and desalted using a 50 mg C18 Sep-Pak cartridge (Waters). The eluted peptides were dissolved in 500 µl of 25 mM NH_4_HCO_3_ and fractionated by high pH reversed phase HPLC. Using an ACQUITY Arc Bio System (Waters), the samples were loaded to a Kinetex 5 µm EVO C18 100 Å LC Column 250×4.6 mm (Phenomenex). Fractionation was performed with the following gradient of solvents A (water), B (acetonitrile), and C (25 mM NH_4_HCO_3_) at a flow rate of 1 ml min^−1^: solvent C was kept at 10 % throughout the gradient; solvent B: 0–5 min: 5 %, 5–10 min: 5–13 % curve 5, 10–70 min: 13–40 %, 70–75 min: 40–50 %, 75–80 %: 50–80 %; followed by 5 min at 80 % B and re-equilibration to 5 % for 24 min. Fractions of 1 ml were collected, dried down, and concatenated to produce on average 25 final fractions for MS analysis.

### Analysis of TMT-labelled samples by LC-MS on an Orbitrap Eclipse Tribrid instrument

Aliquots of all concatenated fractions were analysed by nanoLC-MS/MS on an Orbitrap Eclipse Tribrid mass spectrometer coupled to an UltiMate 3000 RSLCnano LC system (Thermo Fisher Scientific). The samples were loaded onto a trap column (nanoEase M/Z Symmetry C18 Trap Column, Waters) with 0.1 % TFA at 15 µl min^−1^ for 3 min. The trap column was then switched in-line with the analytical column (nanoEase M/Z column, HSS C18 T3, 1.8 µm, 100 Å, 250 mm × 0.75 µm, Waters) for separation using the following gradient of solvents A (water, 0.1 % formic acid) and B (80 % acetonitrile, 0.1 % formic acid) at a flow rate of 0.2 µl min^−1^: 0–3 min 3 % B (parallel to trapping); 3–10 min increase B curve 4–12 %; 10–105 min linear increase B to 47 %; 105–113 min increase B curve 7–99 %; followed 3 min at 99 % B and re-equilibration to 3 % B for 23 min.

Mass spectrometry data were acquired with the following parameters in positive ion mode: MS1/OT: resolution 120K, profile mode, mass range m/z 400–1800, AGC target 100 %, max inject time 50 ms; MS2/IT: data-dependent analysis with the following parameters: IT rapid mode, centroid mode, quadrupole isolation window 0.7 Da, charge states 2–5, threshold 1.9e4, CE=30, AGC target standard, max. inject time 50 ms, dynamic exclusion one count/15 s/±7 ppm; MS3 synchronous precursor selection (SPS): 10 SPS precursors, isolation window 0.7 Da, HCD fragmentation with CE=50, Orbitrap Turbo TMT and TMTpro resolution 30 k, AGC target 200 %, max inject time 105 ms, Real Time Search: protein database *S. coelicolor* A3(2) M145 (uniprot.org, 8126 entries), enzyme trypsin, 1 missed cleavage, oxidation (M) as variable, carbamidomethyl (C) and TMTpro as fixed modifications, Xcorr=1, dCn=0.05. The raw data were processed for protein identification and quantification using the Proteome Discoverer 3.0 (PD3.0) software (Thermo Scientific). A protein fasta sequence database for *S. coelicolor* (8126 entries) was downloaded from uniport.org and used for the database search together with a database for common contaminants (250 entries).

The workflow started with spectrum recalibration. The database search was performed using the chimerys node (MSAID, Munich, Germany) with the inferys_2.1_fragmentation model, enzyme trypsin with one missed cleavage, 0.5 Da fragment tolerance, oxidation (M) as variable modification, carbamidomethyl (C) and TMT10plex (K, N-term) as static modifications. A parallel search was performed using the Comet node with the Comet_version 2019.01 rev. 0 parameter file. For Comet the precursor tolerance was set to 6 ppm, otherwise the same parameters as for chimerys were used. Matches were evaluated using Percolator based on q-values. Reporter ions were extracted using the most confident centroid with 20 ppm integration tolerance. The consensus workflow included the following parameters: only unique peptides (protein groups) for quantification, intensity-based abundance, channel correction values applied (TMT Lot VI306840), co-isolation/SPS matches thresholds 50 %/70 %, normalized chimerys Coefficient Threshold 0.8, normalization on total peptide abundances, protein abundance-based ratio calculation, missing values imputation by low abundance resampling, hypothesis testing by *t*-test (background based), adjusted *P*-value calculation by BH-method. The mass spectrometry proteomics data have been deposited to the ProteomeXchange Consortium via the PRIDE partner repository [23] with the dataset identifier PXD040579.

### Chromatin immuno-precipitation followed by sequencing (ChIP-Seq)

*Streptomyces* strains were inoculated onto cellophane covered DNA and DNAD agar plates as triplicate colonies using 5 µl spores for each and grown for 5 days at 30 °C. To cross-link proteins to DNA the cellophane discs were removed and submersed in 10 ml fresh 1 % (v/v) formaldehyde solution at room temperature for 20 min. After a 5 min incubation in 10 ml 0.5 M glycine the mycelium was harvested and washed twice with 25 ml ice-cold PBS pH 7.4 before being flash frozen in liquid nitrogen and stored at −80 °C. For lysis the pellets were resuspended in 2 ml lysis buffer (10 mM Tris-HCl pH 8.0, 50 mM NaCl, 10 mg ml^−1^ lysozyme, EDTA-free protease inhibitor) before 750 µl was transferred to a 2 ml microcentrifuge tube and samples incubated at 37 °C for 30 min. For fragmentation 750 µl IP buffer (100 mM Tris-HCl pH 8.0, 250 mM NaCl, 0.5 % v/v Triton X-100, 0.1 % SDS, EDTA-free protease inhibitor) was added and samples sonicated on ice at 50 Hz for 20 cycles of 10 s with at least 1 min of rest between each cycle. In total, 25 µl of the crude lysate was combined with 75 µl TE Buffer (10 mM Tris-HCl pH 8.0, 1 mM EDTA) and extracted with 100 µl phenol:chloroform. Overall, 2 µl 1 mg ml^−1^ RNase A was mixed with 25 µl of the resulting extract, incubated at 37 °C for 30 min and run on a 1 % agarose gel to confirm a smear centred at 500 bp. The remaining crude lysate was centrifuged for 15 min at 13 000 r.p.m., 4 °C and supernatant saved. To prepare for binding 750 µl of Anti-FLAG M2 magnetic beads (Sigma Aldrich) were washed in 3.75 ml 0.5 IP buffer. The bead slurry was then mixed with 40 µl clarified lysate and incubated overnight on a vertical rotator. The lysate was removed, and the beads washed four times with 500 µl 0.5 IP buffer including 10 min vertical rotation at 4 °C each cycle. Washed beads were incubated in 100 µl elution buffer (50 mM Tris-HCl pH 7.6, 10 mM EDTA, 1 % SDS) overnight at 65 °C. Eluant was removed and saved before an additional 50 µl elution buffer was added and incubated at 65 °C for a further 5 min. Once removed 3 µl 10 mg ml^−1^ proteinase K was added to the combined eluants and incubated at 55 °C for 2 h. DNA was extracted with 200 µl phenol:chloroform and purified using a QIAquick PCR Purification Kit (QIAGEN) following the manufacturer’s instructions. DNA was eluted in 50 µl Buffer EB (10 mM Tris-HCl pH 8.5) and 47 µl snap frozen in liquid nitrogen and stored at −80 °C. The remaining 3 µl of the sample was used to determine the DNA concentration and quality. The Qubit Fluorometer 2.0 with the high-sensitivity kit was used for DNA concentration whilst DNA purity was quantified using a Nanodrop 2000 UV-Vis Spectrophotometer. The stored DNA samples were the shipped to Novogene on dry ice for sequencing using the NovaSeq 6000 Sequencing System (Illumina).

Raw sequencing data were received as FASTQ files from Novogene and reads were aligned to the relevant reference genome. A normalized local enrichment was calculated along the chromosome by comparing the density of mapped reads in 30 nucleotide windows moving in steps of 15 nucleotides to the density of mapped reads in the 3000-nucleotide region surrounding the window.

### qRT-PCR on *htrA3* and *htrB*

To perform quantitative RT-PCR (qRT-PCR), *S. coelicolor* WT and *∆cutRS* strains were grown on DNA or DNAD agar on top of sterilized cellophane discs at 30 °C for 9 days. Colonies were removed from the cellophanes kept in liquid nitrogen whilstcrushed using a sterile pestle and mortar on dry ice. Crushed samples were resuspended in 1 ml RLT Buffer (Qiagen) supplemented with 1 % 2-mercaptoethanol. This suspension was added to a QIA-shredder column (Qiagen) with the flowthrough transferred to a new 1.5 ml microfuge tube (leaving the pellet). Then, 700 µl acidic phenol:chloroform was added and incubated at room temperature for 3 minutes, and subsequently centrifuged at 13 000 r.p.m. for 20 min. The resulting upper phase was mixed with 0.5 volumes 95 % ethanol. This was then applied to a RNeasy Mini spin column (Qiagen) following the manufacturer’s protocol. The eluant was treated with the Turbo-DNase kit (Invitrogen) followed by a RNeasy mini clean-up kit (Qiagen) both used according to manufacturer’s instructions. The samples were then flash frozen in liquid nitrogen for storage at −80 °C. RNA was quantified with Nanodrop and Qubit Fluorometer and~1 mg converted to cDNA using the LunaScript RT SuperMix Kit (NEB) as per the manufacturer’s instructions, including No-RT reactions. Primers for each transcript were optimized using serial dilutions of template DNA and checked for efficiency by generating standard curves and calculating as follows: *E=(10slope−1)–1*. Primer sets with an efficiency between 90 and 110 % and within 5 % of each other were selected. qRT-PCR was run using the QuantStudio1 (Applied Biosystems) with Luna Universal qPCR Master Mix according to the manufacturer’s guidelines for a 20 µl reaction with 2 µl template cDNA and a final concentration of 0.25 mM of each primer. Controls with no reverse transcriptase were run to ensure the absence of contaminating gDNA. ΔC_T_ values were normalized to the *hrdB* gene, which encodes the housekeeping RNA polymerase sigma factor.

## Results

### CutRS represses actinorhodin production in a glucose-dependent manner

**Fig. 1.**
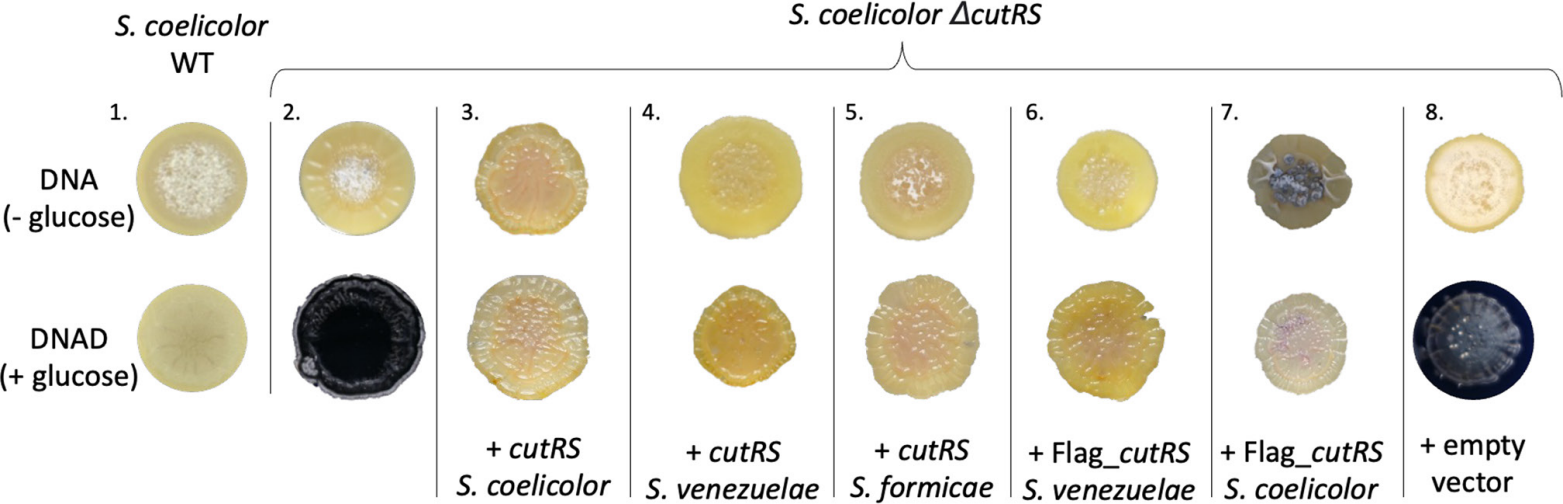
*
S *. coelicolor* ∆cutRS* over-produces the blue antibiotic actinorhodin on growth medium containing glucose and can be complemented by the *cutRS* genes from distantly related species. Agar plate grown cultures of, from left: [1] wild-type *S. coelicolor* M145 [2], *S. coelicolor ∆cutRS* [3], *∆cutRS* containing pIJ10257 with the *S. venezuelae cutRS* operon under the control of the ermE* promoter (ermEp*) [4]; *∆cutRS* containing pIJ10257 with the *S. venezuelae cutRS* operon under the control of ermEp* [5]; *∆cutRS* containing pIJ10257 with the *S. formicae cutRS* operon under the control of the ermEp* [6]; *∆cutRS* containing the *S. venezuelae cutRS* operon under the control of its native promoter and encoding a Flag-tagged CutR [7]; *∆cutRS* containing pIJ10257 with the *S. coelicolor cutRS* operon under the control of its native promoter and encoding a Flag-tagged CutR [8]; *∆cutRS* containing the empty pIJ10257 vector; The plates contain Difco nutrient agar without d-glucose (DNA) or with d-glucose (DNAD) as indicated.

We made a fresh *∆cutRS* mutant in *S. coelicolor* M145 and confirmed that it over-produces the blue-pigmented antibiotic actinorhodin when grown on Difco nutrient agar supplemented with d-glucose (DNAD, Fig. 1). However, there is no obvious growth or developmental phenotype and the *S. coelicolor ∆cutRS* mutant does not visibly produce actinorhodin when grown on Difco nutrient agar without glucose (DNA, Fig. 1). This is surprising because glucose generally represses antibiotic production through glucose-mediated carbon catabolite repression [24]. Given the *cutRS* operon is highly conserved in *Streptomyces* genomes [9] we attempted to complement the *S. coelicolor ∆cutRS* mutant with the *cutRS* genes from *S. coelicolor* and from the more distantly related species *S. venezuelae* [13] and *S. formicae* [12]. In all cases, the introduction of these genes back into the mutant reversed the over-production of actinorhodin suggesting they are complementing the mutation (Fig. 1). The differences in colony morphology observed in Fig. 1 are likely due to the natural biological variation generally observed when culturing *Streptomyces* species.

Next, we compared the proteomes of wild-type *S. coelicolor* and the isogenic *∆cutRS* strain using quantitative tandem-mass-tagged (TMT) proteomics to determine if the actinorhodin biosynthetic pathway is upregulated. The data detected 19 of the 22 of the proteins encoded by the actinorhodin BGC and they are all increased in abundance between 3.5- and 300-fold in the *∆cutRS* mutant relative to the wild-type strain grown on DNAD. Three of the proteins encoded by the actinorhodin BGC were not detected in this experiment (Table 5). When the strains were grown without glucose 17 of the 19 proteins encoded by the actinorhodin BGC were upregulated between two- and tenfold in the *∆cutRS* mutant relative to wild-type whereas levels of the ActAB actinorhodin transporter were lower in the mutant strain (Table 5). ActII-4, the cluster situated activator of this pathway, which activates the expression of the other genes in the BGC, is fivefold higher on DNAD and only threefold higher on DNA whereas the change for ActR, the cluster situated repressor, is less substantial, upregulated 3.6-fold on DNAD and 2.7-fold on DNA. However, glucose is not acting via CutRS (which are absent) and we cannot explain the repressive effect of CutRS on the actinorhodin biosynthesis pathway because CutR does not bind anywhere within the actinorhodin BGC as described in detail below.

**Table 5.** Levels of proteins encoded by the actinorhodin BGC in the *S. coelicolor ∆cutRS* mutant relative to the wild-type strain in the presence (DNAD) and absence (DNA) of glucose

SCO no.	Protein name	Function	DNAD	DNA
			*∆cutRS* vs WT	*∆cutRS* vs WT
SCO5071	ActVI-A	Putative hydroxylacyl-CoA dehydrogenase	134.4	4.5
SCO5072	ActVI-1	Stereospecific ketoreductase	29.5	7.3
SCO5073	ActVI-2	Enoyl reductase	43.8	3.9
SCO5074	ActVI-3	Oxidoreductase	298.6	3.5
SCO5075	ActVI-4	Enoyl reductase	32.7	4.2
SCO5076	ActVA-1	MFS transporter		
SCO5077	ActVA-2	Dimerization of a BIQ intermediate via C-C bond formation?	6.8	2.6
SCO5078	ActVA-3	Involved in C-6 hydroxylation	27.2	7.4
SCO5079	ActVA-4	Regiospecific dimerization via C–C bond formation	86.1	6.4
SCO5080	ActVA-5	Mono-oxygenase (requires ActVB)	37.9	5.2
SCO5081	ActVA-6	Hydroxylase	16.0	2.4
SCO5082	ActR	Repressor of *actAB*	3.6	2.7
SCO5083	ActII-2 (ActA)	Export pump	35.4	0.8
SCO5084	ActII-3 (ActB)	Export pump	24.1	1.9
SCO5085	ActII-4	Transcription activator	4.9	2.8
SCO5086	ActIII	Keto acyl reductase	38.7	5.2
SCO5087	ActI-1	PKS alpha subunit	19.9	6.4
SCO5088	ActI-2	PKS beta subunit	38.8	10.5
SCO5089	ActI-3	PKS acyl carrier protein	22.6	2.9
SCO5090	ActVII	Cyclase / dehydratase	5.9	10.0
SCO5091	ActIV	Cyclase-Thioesterase	12.3	2.6
SCO5092	ActVB	Oxidoreductase	7.3	3.3

### Identifying CutR binding sites on the *S. coelicolor* genome

**Fig. 2.**
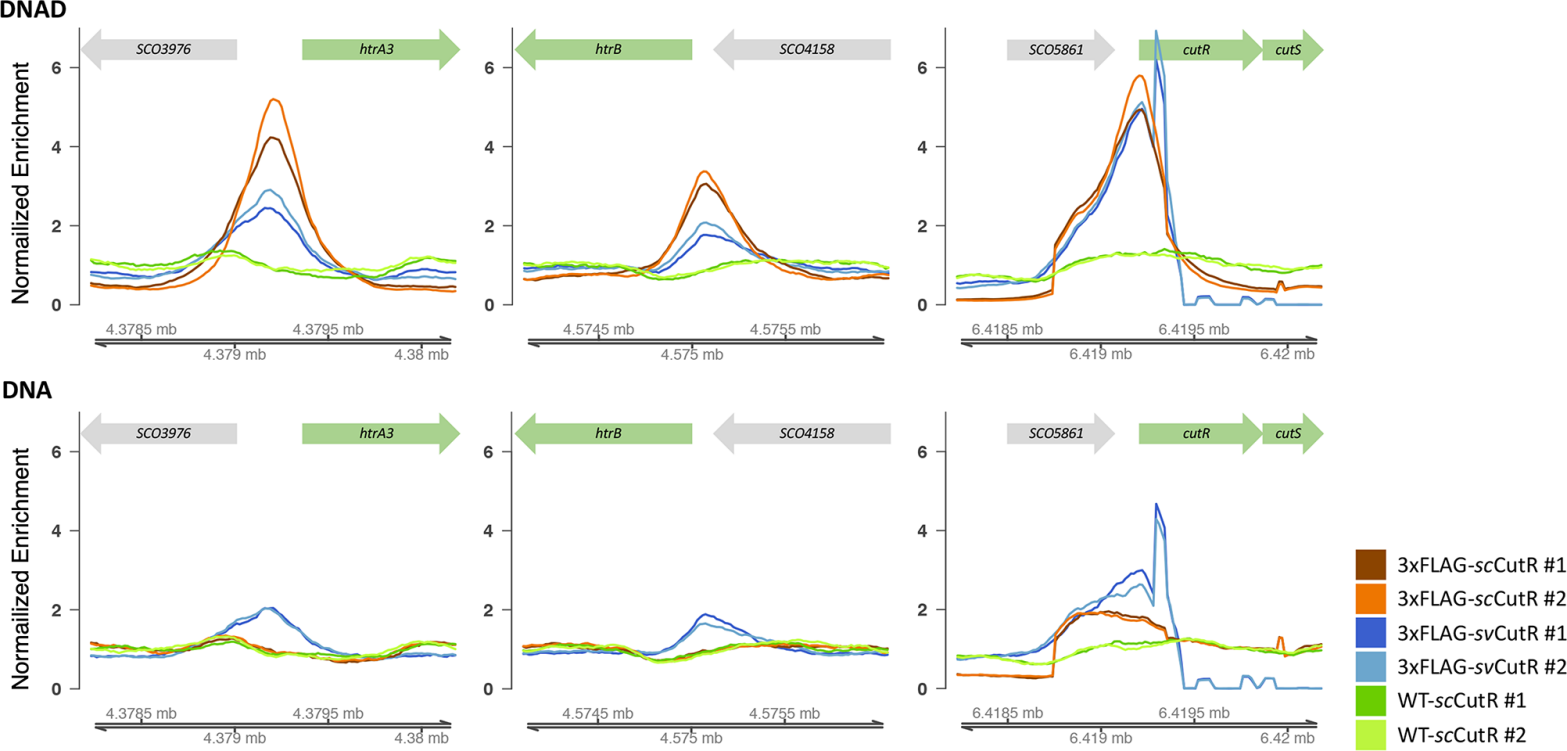
CutR ChIP-seq peaks at the *htrA3*, *htrB* and *cutRS* promoters. ChIP-seq was performed in duplicate against Flag-tagged *S. coelicolor* CutR (scCutR, brown lines) and *S. venezuelae* CutR (svCutR, blue lines) and the wild-type strain with no tag (green lines) grown on Difco Nutrient Agar (DNA) or DNA +D-glucose (DNAD). Binding of CutR to the *htrA3* and *htrB* promoter is enhanced by the presence of glucose in the growth medium.

We complemented the *∆cutRS* mutant with the *SccutRS* operon encoding wild-type CutS and C-terminally 3×Flag-tagged ScCutR (Fig. 1) and then performed ChIP-seq on the Flag-tagged and wild-type (negative control) strains using monoclonal anti-Flag antibodies. The strains were grown on DNA and DNAD plates for 3 days before the mycelium was cross-linked with formaldehyde and harvested for ChIP-seq analysis. For *S. coelicolor* grown without glucose (DNA), the sequence data show there were no significantly enriched targets (>twofold) in the Flag-tagged ScCutR strain relative to the wild-type, whereas in the presence of glucose (DNAD) there were 85 sites of enrichment in the Flag-tagged ScCutR strain (Table S1). Notably, ScCutR did not bind upstream of any genes in the actinorhodin BGC suggesting that ScCutR repression of actinorhodin biosynthesis is indirect. Of the 85 direct target genes, 20 encode hypothetical proteins of unknown function, seven encode putative transcription factors, 38 encode predicted membrane or secreted proteins, 13 are related to primary or secondary metabolism and seven are implicated in protein synthesis. CutRS is highly conserved in the genus *Streptomyces* and the *S. venezuelae cutRS* genes complement the *S. coelicolor ∆cutRS* mutant so we repeated the ChIP-seq in *S. coelicolor* using 3×Flag-tagged SvCutRS (Fig. 1) and identified a core regulon of 16 CutR targets that are bound by both ScCutR and SvCutR in *S. coelicolor* M145 (Fig. 2, Table 6).

**Table 6.** Target promoters enriched in the ChIP-seq experiments by the *S. coelicolor* (*Sc*CutR) and *S. venezuelae* (*Sv*CutR) CutR proteins given as relative enrichment fold change vs control in *S. coelicolor* and their product levels in the mutant versus WT as measured by TMT proteomics. *Only one unique peptide detected

Genome position	Relative enrichment (fold change vs control)				Downstream genes	Annotation	[protein] in *∆cutRS* / WT
	*Sc*CutR DNAD	*Sc*CutR DNA	*Sv*CutR DNAD	*Sv*CutR DNA			
776 415	5.53	1.17	4.19	3.44	*SCO0733*	Hypothetical	nd
1 518 450	5.24	1.07	3.71	2.63	*SCO1422*	Membrane protein	0.12
1 612 020	4.54	0.99	2.76	1.26	*SCO1507*	Putative VKOR protein	0.253
1 876 320	3.97	1.19	2.31	1.84	*SCO1754*	Putative peptidase inhibitor	0.705
2 271 015	4.10	1.26	2.14	2.38	*SCO2112* *SCO2113*	Putative oxidase Bacterioferrin	1.009 nd
3 447 120	3.72	0.98	2.09	1.81	*SCO3146*	Secreted protein	1.5
3 985 875	3.98	1.11	2.06	2.00	*SCO3608* *SCO3609*	Membrane bound serine protease Membrane protein	nd 0.486
4 064 625	5.66	1.35	4.46	4.37	*SCO3681*	Hypothetical protein	nd
4 379 205	4.71	0.94	2.67	2.04	*SCO3977*	HtrA3 protease	0.303
4 575 075	3.21	0.94	1.92	1.77	*SCO4157*	HtrB protease	12.60*
4 710 390	2.92	0.96	1.81	2.09	*SCO4295*	Putative cold shock protein	0.36
5 415 990	4.74	1.09	3.43	3.47	*SCO4978* *SCO4979*	Integral membrane protein Phosphoenolpyruvate carboxykinase	nd nd
5 593 545	5.43	0.90	3.26	3.63	SCO5146 SCO5147	Methyltransferase ECF-subfamily sigma factor	0.409 0.449
6 025 335	4.88	0.98	3.05	2.79	SCO5530	Membrane protein	0.201
6 419 205	4.22	1.56	5.13	3.97	SCO5862-63	CutRS	na
8 072 745	4.08	0.93	2.29	2.35	SCO7261 SCO7262	Membrane protein Hypothetical protein	nd nd

These core targets include the *cutRS* promoter, the promoter upstream of the uncharacterised ECF RNA polymerase sigma factor gene *sco5147*, and eight genes encoding proteins with putative cell envelope functions, including *sco3977* and *sco4157*, which encode the high-temperature requirement proteases HtrA3 and HtrB, respectively. HtrA-like proteins typically act as quality-control proteases and chaperones to monitor secreted protein folding outside the cell and *htrB* is also regulated by the secretion stress sensing two-component system CssRS, which activates its expression and that of the other two conserved *htrA*-like genes in *Streptomyces* species, *htrA1* and *htrA2* [14]. CutR also binds upstream of *sco1507*, which encodes a putative vitamin K epoxide reductase (VKOR), a group of enzymes, which can replace DsbB-like enzymes and recycle the DsbA-family enzymes that catalyse disulphide bond formation in secreted proteins [15].

### CutRS directly controls the production of HtrA3, HtrB and the putative VKOR enzyme SCO1507

**Fig. 3.**
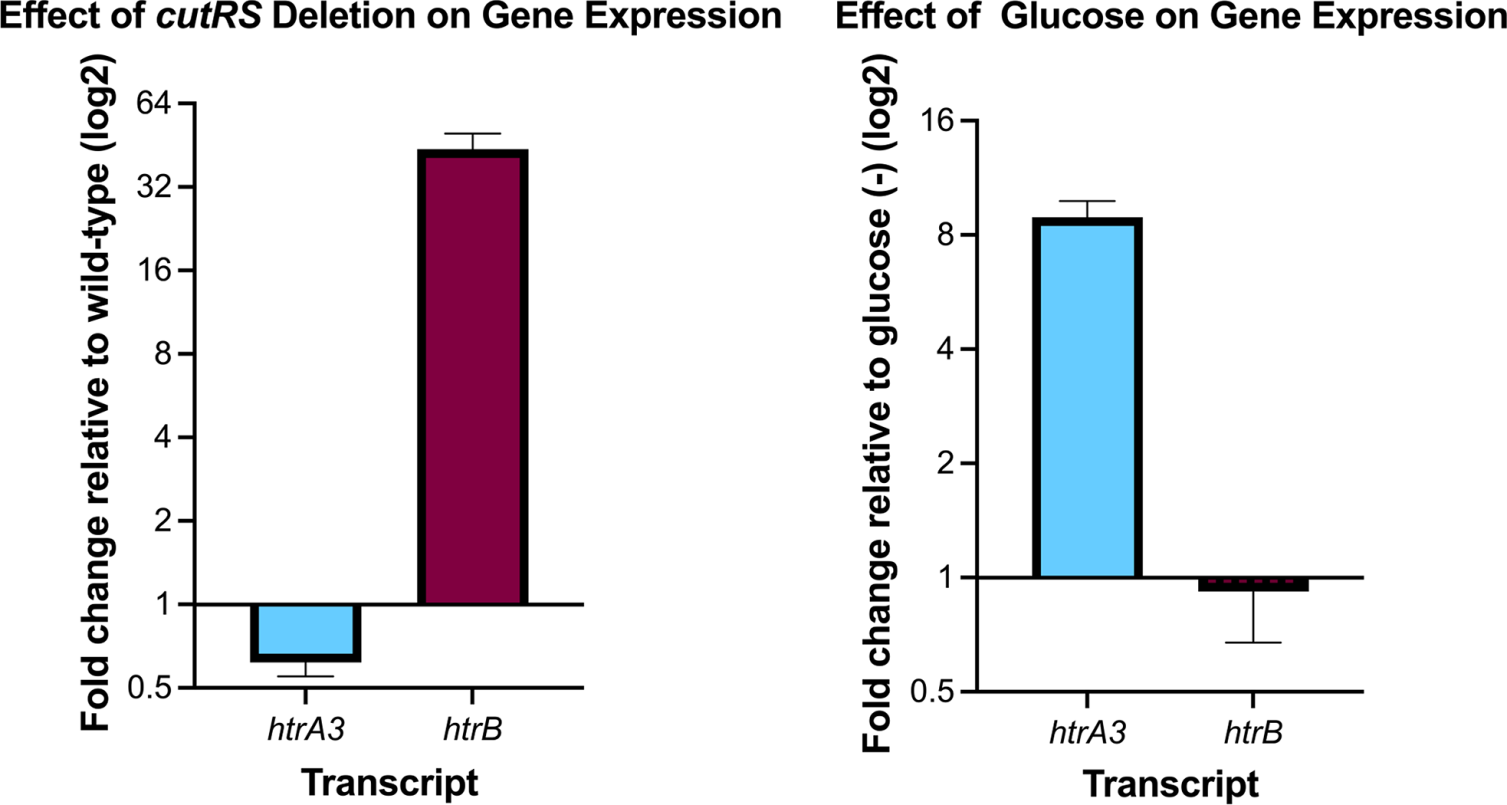
qRT-PCR on *htrA3* and *htrB*. Deletion of *cutRS* results in a 0.6-fold downregulation of *htrA3* and 44-fold increase in htrB expression. Conversely *htrA3* is upregulated in the wild-type strain in the presence of glucose (ninefold) and *htrB* is slightly downregulated (0.9-fold). These data follow the pattern of protein detection under the same conditions using TMT-proteomics. Error bars represent standard deviation across two–four biological and two technical replicates

The TMT proteomics data show that HtrA3 is 3.2-fold higher in wild-type *S. coelicolor* relative to *∆cutRS* while HtrB is 12-fold higher in the *∆cutRS* mutant relative to the wild-type strain (Table 6). Together with the ChIP-seq data this indicates that CutR directly activates expression of *htrA3* and directly represses expression of *htrB* in *S. coelicolor *. To further test this trend of CutR-mediated repression and activation qRT-PCR was carried out and the results confirmed that CutR represses the expression of *htrB* and activates *htrA3* (Fig. 3). The ChIP-seq and proteomics data also show that CutR directly activates production of the VKOR homologue SCO1507, which is 3.75-fold higher in wild-type *S. coelicolor* versus the *∆cutRS* mutant (Table 5). VKOR proteins are involved in the formation of disulphide bonds in secreted proteins by recycling DsbA-like proteins in bacteria that lack DsbB [15]. Consistent with this, *S. coelicolor* encodes a DsbA homologue (SCO2634) but does not encode DsbB, suggesting DsbA might be recycled by VKOR SCO1507. Of the remaining 16 target genes pulled down by both Flag-tagged ScCutR and SvCutR in *S. coelicolor *, nine were either not detected in the proteomics experiment or were not significantly affected by the loss of CutRS, including CutRS which are not present in the *∆cutRS* strain (Table 5). The remaining four CutR target promoters are all activated by CutRS and drive expression of uncharacterized proteins of unknown function. These are the putative FxsA-family membrane protein SCO1422 (8.4-fold), the putative cold shock domain protein SCO4295 (3-fold), the ECF RNA polymerase sigma factor SCO5147 (2-fold) and its divergently encoded O-methyltransferase SCO5146 (2-fold), and the putative membrane protein SCO5530 (5.6-fold).

## Discussion

In this work, we confirmed that loss of CutRS increases the production of the redox active antibiotic actinorhodin in *S. coelicolor* and showed that production of the actinorhodin biosynthetic enzymes is significantly upregulated in a *∆cutRS* mutant. This is glucose dependent with upregulation of the biosynthetic enzymes up to 10-fold on DNA agar with no glucose and up to 300-fold on DNA agar with glucose (DNAD). This likely explains why the ∆*cutRS* mutant visibly over produces actinorhodin on DNAD but not DNA. However, we conclude that CutR-mediated repression of the actinorhodin BGC is indirect because CutR does not bind anywhere within this gene cluster. Instead, the majority of direct CutR targets are membrane or secreted proteins (Table 5 and S1). Furthermore, the combined ChIP-seq and proteomics data show that CutR directly controls, in an antagonistic way, the production of two quality-control proteases named HtrA3 and HtrB [14] that likely play a role in the secretion stress response. Another link to the secretion stress response is the enhanced production of the VKOR homologue SCO1507, which is predicted to act like DsbB and recycle DsbA after it has catalysed disulphide bond formation in secreted proteins [15]. Up to 75 % of *Streptomyces* secreted proteins have two or more cysteine residues, excluding those in lipoprotein signal peptides [25], and it has been proposed that these cysteines play a crucial role in protein folding (via disulphide bond formation) outside the cell. The link here with actinorhodin is tentative but intriguing. VKORs can use quinones as electron acceptors during disulphide bond formation [15]. As a benzoisochromanequinone homodimer, actinorhodin has the potential to act as an alternate electron acceptor for SCO1057 [26]. It is interesting to note that Lejeune *et al*. have proposed that the quinones in actinorhodin can capture excess electrons from reactive oxygen and nitrogen species during oxidative metabolism, thus acting as an anti-oxidant [27].

In summary, we propose that CutRS is likely involved in the secretion stress response and must work alongside the conserved CssRS two component system, which activates the expression of *htrA1*, *htrA2* and *htrB* [14]. Understanding the interplay of these systems, their opposing effects on HtrB production and the roles of the four conserved HtrA-like foldases will be crucial in understanding the bacterial secretion stress response in these bacteria. Furthermore, although the effects of *cutRS* deletion on antibiotic production are indirect, the fact that the *cutRS* mutant makes more actinorhodin in the presence of glucose could also be a useful industrial trait, allowing increased growth and antibiotic yields. Future work will focus on further understanding the interplay of glucose, CutRS activity and antibiotic production in *Streptomyces* species.

## Supplementary Data

Supplementary material 1
